# Structural Organization of Human Full-Length PAR3 and the aPKC–PAR6 Complex

**DOI:** 10.1007/s12033-022-00504-1

**Published:** 2022-05-24

**Authors:** Le T. M. Le, Srdja Drakulic, Jens R. Nyengaard, Monika M. Golas, Bjoern Sander

**Affiliations:** 1 Core Center for Molecular Morphology, Section for Stereology and Microscopy, Department of Clinical Medicine, Aarhus University, Aarhus, Denmark; 2 Department of Pathology, Aarhus University Hospital, Aarhus, Denmark; 3 Department of Biomedicine, Aarhus University, Aarhus, Denmark; 4 Centre for Stochastic Geometry and Advanced Bioimaging, Aarhus University, Wilhelm Meyers Allé 3, Building 1233/1234, 8000 Aarhus C, Denmark; 5 Human Genetics, Faculty of Medicine, University of Augsburg, Stenglinstrasse 2, 86156 Augsburg, Germany; 6 Present Address: Institute of Pathology, Hannover Medical School, Carl-Neuberg-Str. 1, 30625 Hannover, Germany; 7 Present Address: The Hormel Institute, University of Minnesota, Austin, MN USA

**Keywords:** PAR3, PAR6, aPKC, PAR complex, Polarity, Single-particle electron microscopy

## Abstract

The tripartite partition defect (PAR) polarity complex, which includes the proteins PAR3, atypical protein kinase C (aPKC), and PAR6, is a major regulator of cellular polarity. It is highly conserved and expressed in various tissues. Its largest component, PAR3, controls protein–protein interactions of the PAR complex with a variety of interaction partners, and PAR3 self-association is critical for the formation of filament-like structures. However, little is known about the structure of the PAR complex. Here, we purified non-filamentous PAR3 and the aPKC–PAR6 complex and characterized them by single-particle electron microscopy (EM). We expressed and purified an oligomerization-deficient form of PAR3, PAR3_V13D,D70K_, and the active aPKC–PAR6 dimer. For PAR3, engineering at two positions is sufficient to form stable single particles with a maximum dimension of 20 nm. aPKC–PAR6 forms a complex with a maximum dimension of 13.5 nm that contains single copies of aPKC. Thus, the data present a basis for further high-resolution studies of PAR proteins and PAR complex formation.

**Supplementary Information:**

The online version contains supplementary material available at 10.1007/s12033-022-00504-1.

## Introduction

Polarity is a hallmark of cellular development such as anterior–posterior polarity in zygotes, apical-basal and planar cell polarity in epithelia, axon-dendrite differentiation in neurons, and transient polarity in migrating cells [1–7]. Polarity is established and regulated by a set of evolutionarily highly conserved proteins including a set of PAR (for partitioning defective) proteins. PAR proteins have been originally identified as factors required for polarization in *C. elegans* zygotes [8]. The PAR protein family includes a polarity complex (PAR complex) comprising PAR3, atypical protein kinase C (aPKC), and PAR6 [9, 10]. In vertebrates, the PAR complex has been studied extensively in epithelia, where the proteins localize in the apical compartment near tight junctions and have been demonstrated to be central for the establishment and maintenance of apical-basal polarity [11]. The PAR complex is also critical for neural development, where it has been suggested to be essential for the differentiation of neurites into dendrites and the axon [12–14]. Moreover, growing evidence suggests a link between deregulation of PAR3 and cancer development favoring cell proliferation, epithelial–mesenchymal transition (EMT) and metastatic spread in a number of tumor types [15].

In human, its major component, PAR3, is a large (> 1300 amino acids) protein rich in interaction domains. PAR3 binds PAR6 through PDZ (PSD-95, Discs-large, ZO-1) domains [9], and interacts with aPKC through an aPKC-binding domain [16], while PAR6 and aPKC interact with each other through their PB1 (Phox and Bem1) domains [17]. PAR3 has been suggested to bind to two PAR6 proteins via two of its three PDZ domains [18]. The first PDZ domain of PAR3 can also bind to membrane proteins including junctional adhesion molecules (JAM) and the p75 neurotrophin receptor [19–21].

Moreover, PAR3 exhibits a microtubule binding and bundling activity [22], and the PAR complex has been implicated in the regulation of the microtubule and actin cytoskeleton as a critical step in neuronal development [12, 13, 23]. The semi-CRIB (Cdc42- and Rac-interactive binding) domain of PAR6 can bind to the active Rho GTPase CDC42 [9], which activates aPKC to phosphorylate PAR3 and cause PAR3 dissociation from the PAR complex [24].

Major attention has been paid to the ability of the proteins to enrich in certain cellular compartments as a hallmark of polarization [13, 14, 25–27]. Particularly, PAR3 self-association may represent the molecular basis for the enrichment of the PAR complex at target sites. The first approximately 83 amino acids of PAR3 form an N-terminal domain (NTD) that has been demonstrated to exhibit a critical role in self-association of PAR3 [25, 28]. Crystallization and cryo-EM studies of the rat PAR3 NTD fragment have shown that the isolated NTD forms protein helices with a regular pitch through a number of residues including T4, V13, and D70 providing lateral interactions, as well as R9 providing longitudinal interactions [29]. Mutation studies of the isolated PAR3 NTD furthermore suggested that mutations of V13 and D70 prevent oligomerization of NTD fragments [29]. However, the structural organisation of PAR3 clusters in vivo remains unknown.

Although the ability of the PAR complex to function in diverse cellular contexts such as epithelia and neurons would clearly be explained by knowing its structure, still little is known about its architecture. Here, we aimed at characterizing non-polymerized PAR complexes, the heterodimeric aPKC–PAR6 complex, and PAR3 alone.

## Results

### Identification of *PAR3*, *aPKC*, and *PAR6* Isoforms Expressed in Human Neural Cells

**Fig. 1:**
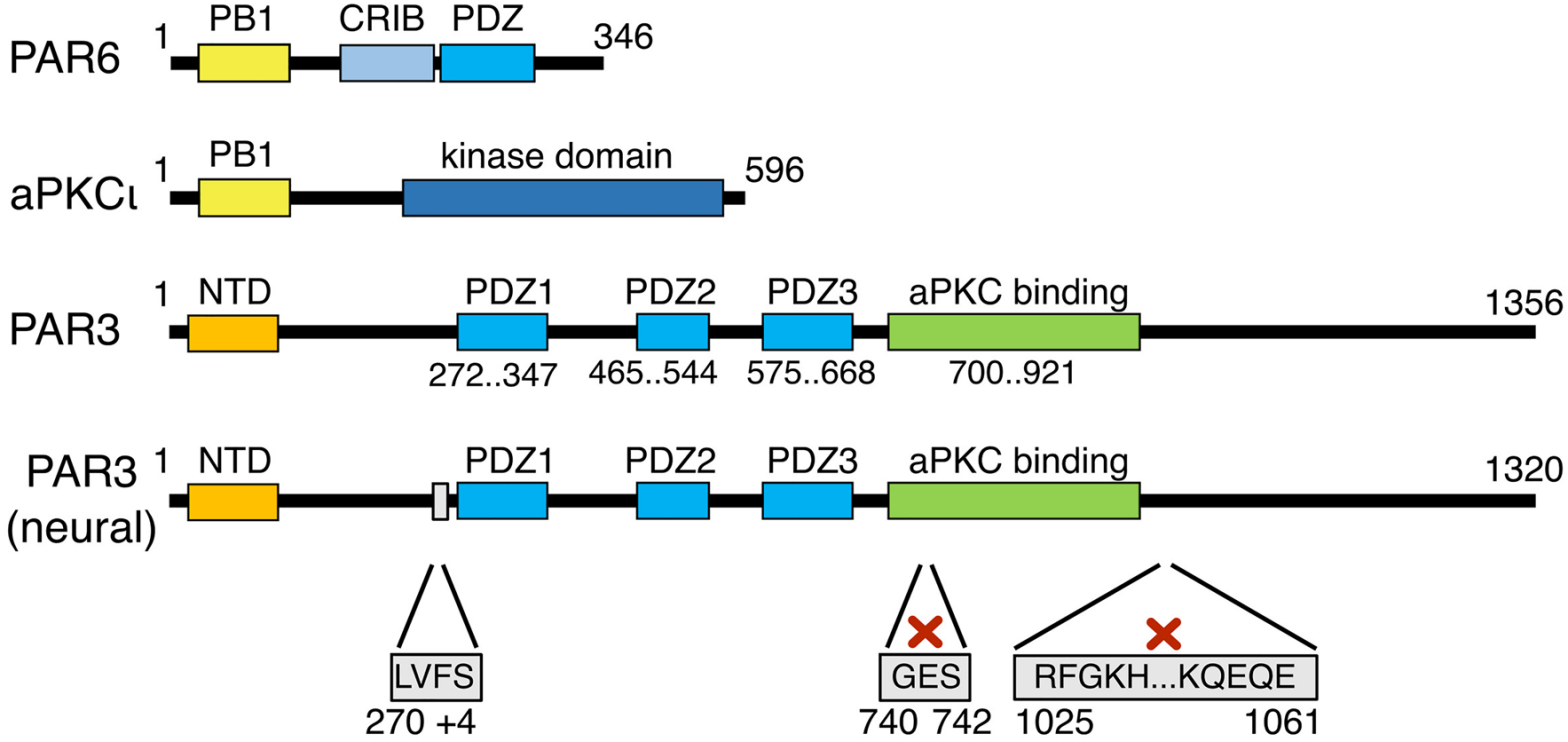
Protein domains of human PAR6, aPKC, and PAR3. PAR6 and aPKC interact via their PB1 domains. aPKC harbors a kinase domain that can phosphorylate PAR3 followed by PAR3 release from the complex. PAR3 binds to PAR6–aPKC via a PDZ/aPKC-binding domain. The neural isoform analyzed herein showed alternative splicing leading to an extra mini-exon at amino acid (aa) 270, an omission of 3 amino acids at aa 739–743, and a skipped exon encoding aa 1024–1062 (positions compared to the canonical PAR3 sequence, accession no. NP_062565.2)

The genes of *PAR6, PAR3*, and *aPKC subtype iota* were amplified from human neural cells [30], and their identity was confirmed by sequencing (Fig. 1). The sequences of the encoded aPKC and PAR6 proteins are identical to the canonical human isoforms (PKCι, GenBank accession code: NP_002731.4; PAR6α; GenBank accession code: NP_001032358.1), respectively. PAR3 is a novel isoform as a result of minor changes in alternative splicing. In comparison to the human PAR3 isoform 1 (GenBank accession code: NP_062565.2), the following differences were observed: (i) the neural PAR3 isoform possessed four additional amino acids after residue 269 with D269 changed to E immediately N-terminal to the PDZ1 domain; (ii) three amino acids were omitted between positions 739–743 in the aPKC-binding domain; and (iii) 37 residues were omitted between positions 1024–1062 C-terminal to the aPKC-binding domain. The omission of these 37 residues is also seen in isoform 4 of PAR3 (NCBI accession no. NP_001171716). All these modifications in the PAR3 isoform can be explained by alternative splice sites of the transcript. Besides, we observed a K312R change in the PDZ1 domain.

### aPKC and PAR6 form a Defined Complex

**Fig. 2:**
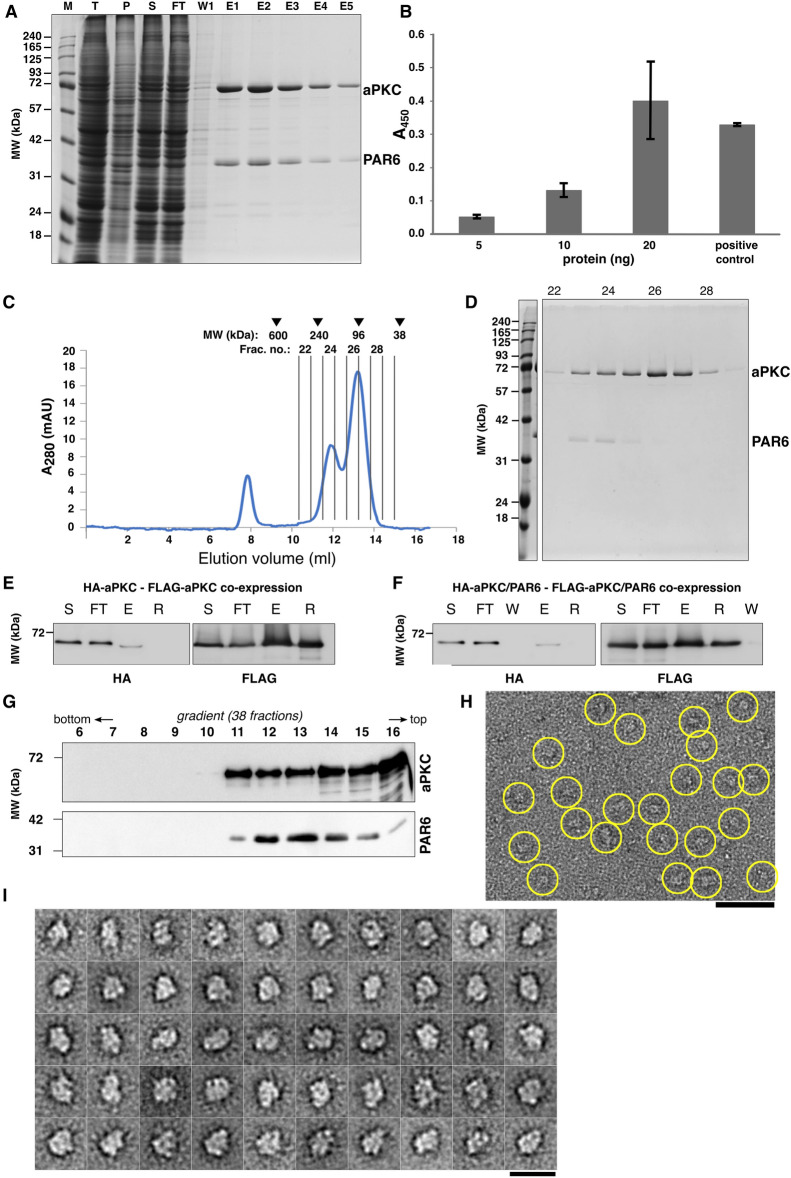
aPKC and PAR6 associate as a heterodimeric complex functional in phosphorylation. **A** Anti-FLAG-affinity selected aPKC–PAR6 complex visualized by SDS-PAGE and Coomassie staining. Proteins are indicated to the right. M, T, P, S, FT, W1, and E1-E5 correspond to marker, total cell lysate, pellet, supernatant, flow through, first wash, and elution fractions 1–5, respectively. **B** Kinase activity of the purified aPKC–PAR6 complex measured as absorption at 450 nm for protein concentrations of 5 ng, 10 ng, and 20 ng, and a positive control (*n* = 3). As positive control, 36 ng of control protein was used. **C** Gel-filtration chromatography profile of the aPKC–PAR6 complex. Protein fractions (500 µl) were collected, and the absorption was monitored at 280 nm wavelength. Running behaviors of standard proteins (in kDa) are indicated at the top. **D** The peak fractions 22–29 (fraction numbers indicated in C) are visualized by a Coomassie-stained SDS-PAGE (theoretical MW: aPKC, 71 kDa; PAR6, 37 kDa). The aPKC–PAR6 complex peaks in fraction 24, and aPKC alone peaks in fractions 26. Proteins are indicated to the right. **E, F** Recovery of HA-aPKC in FLAG pulldown assays upon 3 × FLAG-aPKC,HA-aPKC co-expression (E) and 3 × FLAG-aPKC,HA-aPKC,PAR6 co-expression (F) (S, supernatant; FT, flow through; E, eluate; R, FLAG affinity resin; W, wash). **G** Western blot of the aPKC–PAR6 heterodimer after sedimentation in a 5–20% glycerol gradient. Protein samples were collected in a total of 38 fractions, of which fractions 6–16 are shown (fraction numbering from the bottom; molecular weight marker indicated on the left). **H** A representative negative stain EM image of the aPKC–PAR6 complex using peak fractions 12–14 shows a monodisperse particle population (scale bar: 50 nm). **I** 2D class averages of aPKC–PAR6 demonstrate particles with a compact, moderately elongated shape and maximum dimensions of 13.5 nm (scale bar: 20 nm)

Wild-type tagged aPKC and untagged PAR6 were co-expressed in their full-length forms using a baculovirus/insect cell system. Upon immuno-purification of aPKC via its N-terminal 3 × FLAG tag (Fig. 2A), a dimeric complex was obtained. We confirmed by using a kinase assay that the purified aPKC–PAR6 complex was functionally active in phosphorylation (Fig. 2B). By size exclusion chromatography (SEC), the aPKC–PAR6 complex (theoretical molecular weight of 106 kDa assuming a 1:1 complex) and co-eluting unbound aPKC (theoretical molecular weight of monomer, 68 kDa) showed peaks in fraction 24 and 26, respectively, at elution volumes somewhat higher than expected for near spherical proteins (Fig. 2C, D).

### aPKC Does Not Exhibit Self-Interaction

**Fig. 3:**
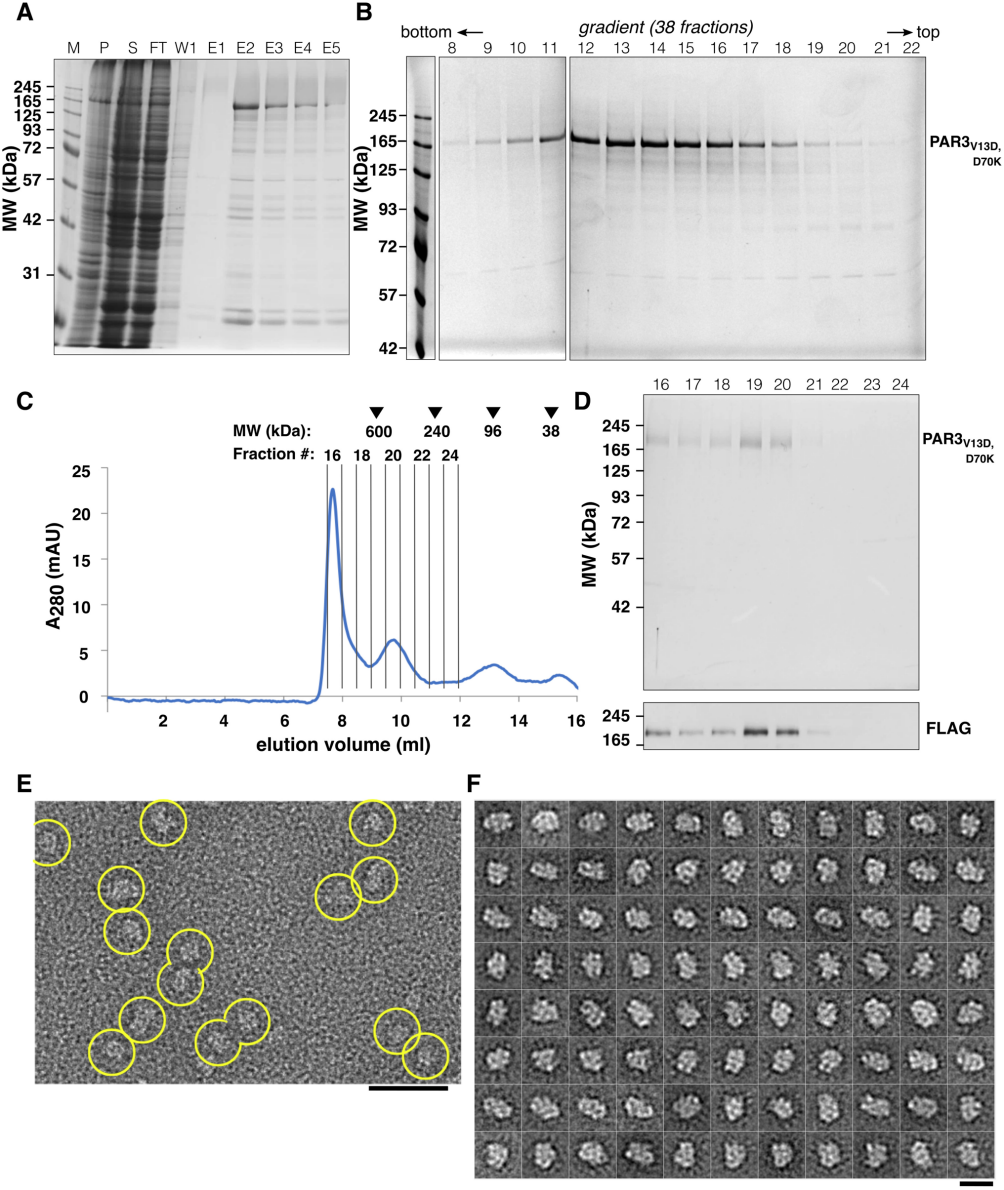
PAR3_V13D,D70K_ is stable in solution and forms elongated particles. **A** Affinity selection of N-terminal 3 × FLAG-tagged PAR3_V13D,D70K_ visualized by Coomassie-stained SDS-PAGE (M, marker; P, pellet; S, supernatant; FT, flow through; W1, wash fraction 1; E1-E5, elution fraction 1–5). **B** 5–20% glycerol gradient fractionation of PAR3_V13D,D70K_ as visualized by Coomassie-stained SDS-PAGE. Shown are fractions 8–22 out of 38 fractions in total. PAR3_V13D,D70K_ forms a defined peak on the gradient. **C** In SEC, PAR3_V13D,D70K_ peaks in fractions 19 and 20. Position of calibration proteins (in kDa) and fractions used for SDS-PAGE analysis (D) are indicated at the top. **D** SEC fractions as indicated in C are separated by SDS-PAGE and visualized by Coomassie staining (top). The presence of PAR3_V13D,D70K_ was confirmed by anti-FLAG western blot (bottom). **E** Single particles observed by negative stain EM. **F** Representative 2D class averages of PAR3_V13D,D70K_ showing particles up to 20 nm in diameter. The scale bars correspond to 50 nm **E** and 20 nm **F**, respectively

To assess whether or not the main component of the heterodimeric aPKC–PAR6 complex, aPKC occurs in monomeric form, we co-expressed aPKC with two different tags by replacing the *3* × *FLAG* sequence in the *aPKC* construct with an *HA* tag sequence, followed by co-expression of the two distinguishable tagged forms of aPKC and purification via the 3 × FLAG-tag. We expressed HA-aPKC together with 3 × FLAG-aPKC in the absence of PAR6, and we also expressed HA-aPKC and 3 × FLAG-aPKC in the presence of PAR6 to test whether the presence of PAR6 had an influence on the stoichiometry of the protein complex (Supplementary Fig. S1A, B). We measured the recovery of HA-aPKC in FLAG pulldown assays upon 3 × FLAG-aPKC,HA-aPKC co-expression (Fig. 2E) and 3 × FLAG-aPKC,HA-aPKC,PAR6 co-expression (Fig. 2F) using anti HA western blotting and anti FLAG western blotting as control. Only a minor fraction of HA-aPKC was recovered irrespective of the absence or presence of PAR6: when normalized to the input (supernatant), the elution yielded 0.5% recovery of HA-aPKC and 0.14% of HA-aPKC–PAR6 (Fig. 2E, F; for SDS-PAGE, see Fig. S1A, B) consistent with no noteworthy self-interaction and predominantly monomeric aPKC alone and in complex with PAR6. Thus, the predominant form of the aPKC–PAR6 complex is an assembly with a single copy of aPKC.

### aPKC–PAR6 Forms Moderately Elongated Particles

For all particles, we employed gradient ultracentrifugation as final purification step, which provides an optimum sample quality for EM [31]. When aPKC–PAR6 was run on a 5–20% glycerol gradient, the peak of the protein complex (Fig. 2G and Supplementary Fig. S1C) occurred in fraction 12 – 14 (out of 38 fractions), which corresponds to an apparent Svedberg value of about 4.5S. By EM, raw images showed a monodisperse particle population (Fig. 2H). The maximum dimension of the particles is approximately 13.5 nm, and class averages with about 24 images per class show well-discernible fine-structural details indicating a well-defined structure (Fig. 2I). In particular, the particles reveal an asymmetrical, compact, moderately elongated structure.

We also combined the Stoke’s radii and sedimentation data to a molecular weight estimate (MW) using Erickson’s approximation $MW=4.205 (S\bullet {R}_{S})$ [32] based on Siegel and Monty [33], where *S* is the sedimentation in Svedberg Units and *R*_*s*_ is the radius in nm. For the aPKC–PAR6 complex, this estimation yields a predicted MW of max. ~ 100 kDa (Table 1), which is consistent with a monomeric stoichiometry of the largest protein in the complex, aPKC, in addition to PAR6.

**Table 1:** Characteristics of the protein complexes

	PAR3_V13D,D70K_	aPKC	aPKC–PAR6
Theoretical monomeric MW [kDa]	141	71	108
SEC R_s_ [nm]	6.1	4.0	4.7
Sedimentation coefficient [S]	5.2–7.1	3.5–4.2	4.4–5.3
EM max. diameter [nm]	20	N/A	13.5
MW_SM_ [kDa]	133–182	59–71	87–105

*MW* molecular weight, *SEC* size exclusion column, *R*_*s*_ Stoke’s radius measured by SEC, *S* Svedberg, *EM* electron microscopy, *MW*_*SM*_ molecular weight estimate derived from R_s_ and S using the Siegel-Monty estimation

### PAR3_V13D,D70K_ Forms a Stable Elongated Particle

Initial expression tests of wild-type PAR3 alone indicated that wild-type PAR3 was not stable after elution (data not shown); thus, we investigated a mutant form of PAR3. To this end, we expressed a form of PAR3 mutated at two positions (V13D and D70K) that was reported earlier [29]. Expression tests with PAR3_V13D,D70K_ alone (*i.e.*, not in complex with PAR6–aPKC) showed that PAR3_V13D,D70K_ was stable, and protein degradation could be minimized (Fig. 3A,B), which provided the ability to purify PAR3_V13D,D70K_ in amounts sufficient for EM analysis. In SEC, PAR3_V13D,D70K_ peaked in fraction 19–20 at an elution volume corresponding to a Stokes radius of ~ 6.1 nm, separate from void (Fig. 3C,D and Table 1). Another UV peak (fraction 16) visible nearby the void volume, however, contained smaller amounts of PAR3 as evinced by western blotting (Fig. 3D). PAR3_V13D,D70K_ was subsequently run on a 5–20% glycerol gradient (Fig. 3B), where it peaked around fraction 12–13 out of 38 fractions corresponding to the ~ 7S region (approximate *MW* 133–182 kDa, compare Table 1). These results are consistent with a monomeric protein given the theoretical molecular weight of 141 kDa. EM images of PAR3_V13D,D70K_ showed monodisperse, moderately elongated single particles (Fig. 3E), and the 2D class averages of PAR3_V13D,D70K_ confirmed a well-defined structure with compact shape and maximum dimensions of ~ 20 nm (Fig. 3F).

## Discussion

Herein, we aimed at characterizing the non-polymerized building blocks as smallest units of the PAR complex that is formed by the PAR proteins PAR3, aPKC, and PAR6. To avoid polymerization of PAR3, we took advantage of two point mutations in the NTD of PAR3, V13D, and D70K. These mutations had been previously reported to prevent the PAR3 NTD from self-association [28], and were described to abolish interactions in the isolated NTD fragment of rat Par3 by preventing lateral packing into a helix [28, 29].

The sedimentation and SEC data we present herein are in favor of single copies of the largest subunit, PAR3, and of aPKC and PAR6 in the aPKC–PAR6 complex. For the aPKC–PAR6 complex, the presence of a single aPKC protein within the aPKC–PAR6 complex has independently been validated by the double tagging assay. In the aPKC–PAR6 heterodimer, aPKC and PAR6 interact via the PB1 domain present in both proteins [17]. In the crystal structure, the PB1 domains of aPKC and PAR6 form an asymmetric heterodimer, with one copy of the aPKC and PAR6, each [17]. Together with our pulldown data, these data support a 1:1 stoichiometry of aPKC and PAR6 as smallest unit.

It has been shown that PAR3 binds essentially two copies of PAR6 via its PDZ1 and PDZ3 domains, albeit at different affinities [18]. Both dissociation constants were however reported in the micromolar range [18]. Whether or not the local enrichment of the PAR proteins at the plasma membrane is sufficient to facilitate recruitment of two PAR6 copies (or two aPKC–PAR6 heterodimers) to the same PAR3 proteins under in vivo conditions at the cell membrane, will thus need further investigation.

By EM, we did not observe formation of specific multimers of the PAR3 protein, indicating that higher order assemblies that may have formed despite the engineering were not sufficient for visualization by EM. Further research will be required to investigate how the basic building blocks characterized here enrich into higher order PAR complex assemblies inside the cell. Especially, how the rather large PAR3 protein assembles into higher order complexes and whether or not PAR3 will adopt a helical assembly in vivo remains to be investigated. Future high-resolution cryo-EM reconstructions of the PAR complex based on these data are required to address these questions. Likewise, how the occurrence of alternative splicing of PAR3 shown here contributes to tissue-specific variants of the PAR complex will require more investigation. Overall, our current studies provide projection structures of the PAR components PAR3 and aPKC–PAR6 as a step toward a detailed structural and functional understanding of these components in the establishment and maintenance of cellular polarity.

## Materials and Methods

### Amplification of mRNA from Human Neural Cells

Full-length human *PAR6, PAR3,* and *aPKC subtype iota (PKCɩ)* were amplified with appropriate primers (Supplementary Table S1) and cDNA synthesized using mRNA derived from human neural cells as described previously [30]. The Maxima H minus first strand cDNA synthesis kit (ThermoFisher Scientific, Waltham, MA, U.S.A.) was used for cDNA synthesis. The vector pUC57 (ThermoFisher Scientific) was used to insert the DNA fragments using suitable restriction enzymes.

### Plasmid Construction

*PAR3* was subcloned into the vector pGS-BacA-21122 [34], a derivative of pACEBac1, which introduced a 3 × FLAG to the N-terminus of the expressed protein. *PAR3* was studied as wild-type protein and as an engineered PAR3_V13D,D70K_ with two amino acid changes, V13D and D70K, in the NTD. We introduced a mutation causing a kinase-dead mutant in mouse [35], PKCι_K283R_, into the human gene upon sequence alignment of the human *PKCι* (GenBank accession code: NM_002740.5) and mouse *PKCι* (GenBank accession code: BC021630.1) [35]. The QuickChange Lightning Site-Directed Mutagenesis kit (Agilent Technologies, Santa Clara, CA, U.S.A.) was used for site-directed mutagenesis. By Cre recombination, composite bacmids containing *PAR3*, *PKCι*, and *PAR6* were created from acceptor and donor plasmids as described previously [36] for multi-protein expression in insect cells using the Multibac system [37, 38].

To study the dimeric aPKC–PAR6 complex, the coding sequence of *aPKC* was ligated into the acceptor vector pGS-BacA-21122 [34], and PAR6 was ligated into the donor vector pIDC. Furthermore, the *3* × *FLAG* tag from the *aPKC* plasmid was replaced by an *HA* tag to investigate aPKC self-oligomerization. The plasmids coding for *PKCι* and *PAR6* were combined by Cre-LoxP reactions as outlined [36]. The plasmid constructs created in this study are listed in Supplementary Table S2. The sequence of relevant plasmid elements was confirmed by Sanger sequencing (Eurofins, Ebersberg, Germany or Macrogen Europe, Amsterdam, The Netherlands).

### Protein Expression and Purification

The bacmid and virus preparations for PAR protein expression were performed as described previously [39]. High Five (BTI-TN-5B1-4) or Sf9 cells (both cell lines purchased from ThermoFisher Scientific) were infected with baculovirus carrying *aPKC–PAR6* and *PAR3*_V13D,D70K_, respectively*,* and grown for 72 h. The cells were resuspended in lysis buffer (20 mM HEPES, pH 7.6; 10% glycerol; 400 mM NaCl; 1 mM EDTA; 1 mM PMFS (Sigma-Aldrich, St. Louis, MO, U.S.A.) supplemented with protease inhibitor (Complete ULTRA tablets, EDTA-free; Roche, Mannheim, Germany)). The cells were pelleted, and the supernatant was mixed with equilibrated anti-FLAG resin (100 µl; M2 anti-FLAG affinity gel, Sigma) and incubated at 4 °C for 3 h. After incubation, the sample was centrifuged at 700 × *g* for 10 min to remove the supernatant. The resin was washed three times with washing buffer (20 mM HEPES, pH 7.6; 5% glycerol; 400 mM NaCl). The resin was then transferred into a filter column (M105035F; MoBiTec, Göttingen, Germany) and centrifuged at 100 × *g* for 3 s. The resin was incubated with 100 µl FLAG-elution buffer (20 mM HEPES–NaOH, pH 7.6; 400 mM NaCl; 1 mM EDTA; 1 mM PMFS; 1 × protease inhibitor; 125 µg/ml 3 × FLAG peptide) for 30 min prior to elution as fraction E1. Fraction E2 was eluted as described above with 400 µl FLAG-elution buffer. A volume of 100 µl elution buffer (20 mM HEPES–NaOH pH 7.6, 400 mM NaCl, 1 mM EDTA, 1 mM PMFS, protease inhibitor) was then added to collect fraction E3. This step was repeated three times to elute the remaining proteins as fractions E4, E5, and E6. BCA assays were used to quantify protein amounts.

### Size Exclusion Chromatography

The purified proteins were subjected to SEC on a Superdex 200 column (GE Healthcare, Little Chalfont, U.K.) for the dimeric aPKC–PAR6 and PAR3_V13D,D70K_ in 20 mM HEPES (pH 7.6) supplemented with 400 mM NaCl. The Gel Filtration HMW Calibration Kit (GE Healthcare) was used for calibration of the SECs. For calibration of the elution volume as a function of Stoke’s radius R_s_, R_s_ values reported in [32] were used.

### Gradient Ultracentrifugation

The purified proteins were run in a 5–20% glycerol gradient for 17 h at 4 °C (20 mM HEPES, pH 7.6; 400 mM NaCl) at 352,996 × *g* for the dimeric aPKC–PAR6 complex or 274,824 × *g* for PAR3_V13D,D70K_. The gradients were fractionated into 38 fractions with 5 drops per fraction by fractionation from the bottom of the gradient using a P-1 peristaltic pump (GE Healthcare) as described previously [40﻿]. For estimation of the apparent sedimentation coefficients (S), commercial standards were used (Sigma). As glycerol gradient peaks typically span over multiple fractions, an apparent S value range is given for all particles. Proteins were visualized by Coomassie staining and verified by western blotting.

### Western Blot Analysis

The protein samples were added to SDS loading dye, heated to 95 °C, separated by SDS-PAGE and transferred to a nitrocellulose membrane (ThermoFisher Scientific). The antibodies anti-FLAG M2 (F1804; Sigma-Aldrich, 1:1000), anti-HA (Santa Cruz, sc-805, 1:200), anti-PKC (sc-216, Santa Cruz, 1:500), and anti-PAR6 (sc-33898, Santa Cruz, 1:500) were used as primary antibodies, anti-mouse IgG-Peroxidase (Sigma-Aldrich, 1:10,000), anti-rabbit IgG-Peroxidase (Sigma-Aldrich, 1:5000), and anti-goat IgG-Peroxidase (Sigma-Aldrich, 1:5000) for PAR3, aPKC, and PAR6, respectively, as secondary antibodies. The membranes were developed using SuperSignal West Pico or Femto Chemiluminescent Substrate (ThermoFisher Scientific). The detection was done by an ImageQuant LAS4010 system (GE Healthcare). The images were analyzed by ImageQuant TL toolbox version 8.1 following the company’s instructions and quantified by ImageStudio Lite (LI-COR, Lincoln, NE, U.S.A.).

### EM Image Acquisition

A volume of glutaraldehyde (Sigma-Aldrich) corresponding to a final concentration of 0.075% was added to the protein samples followed by incubation overnight at 4 °C before grid preparation. Negative staining samples were prepared using the sandwich carbon method with home-made carbon film and uranyl formate or uranyl acetate (2%) [41]. The images were taken in a Tecnai T12 electron microscope (FEI, Eindhoven, The Netherlands) with a Multiscan 794 CCD camera (Gatan, Pleasanton, U.S.A.) operated at 120 kV at a nominal magnification of 52,000 × , which corresponded to an apparent magnification of 63,160x. The pixel size on the specimen level was 3.8 Å/pixel.

### EM Image Processing

The particles on the images were selected manually. Determination of defocus and astigmatism of the EM images was done by fitting contrast transfer function (CTF) curves to the power spectra of the images [42]. The particle images were extracted, corrected for CTF-effects, and merged. The data set characteristics are summarized in Table S3. The classification and averaging of particles followed standard methods [43] and were performed in the statistical framework R [44] with 3–10 rounds of particle alignment followed by principal component analysis and unbiased classification using hierarchical ascendant and k-means classification (Table S3).

### PKC Kinase Activity

The activity of purified dimeric aPKC–PAR6 complex was tested using the PKC Kinase Activity Assay Kit (Abcam, Cambridge, U.K.) with a protein concentration dilution series of 5 ng, 10 ng, and 20 ng of the purified protein complex following the manufacturer’s instructions, and the standard error of the mean (SEM) was used for visualization (*n* = 3 replicates). As positive control, 36 ng of control protein (Abcam) was used. A multimode plate reader (EnSpire, PerkinElmer, Waltham, MA, U.S.A.) was used to measure the reaction signal.

## Supplementary Information

Below is the link to the electronic supplementary material.

Supplementary file1 (DOCX 934 kb)

## Data Availability

Additional data are given in the Supporting Material.
