# Supplementary material for: Structural Organization of Human Full-Length PAR3 and the aPKC–PAR6 Complex
Source: Mol Biotechnol. 2022 May 24;64(12):1319–27. doi: 10.1007/s12033-022-00504-1 (PMC9573856; doi:10.1007/s12033-022-00504-1)
Supplement: Supplementary file 1 — Supplementary file1 (DOCX 934 kb) [file 12033_2022_504_MOESM1_ESM.docx]

# Structural organization of human full-length PAR3 and the aPKC-PAR6 complex

Le T. M. Le^1,6^, Srdja Drakulic^2^, Jens R. Nyengaard^1,3^, Monika M. Golas^2,4^, and Bjoern Sander^3,5,*^

^1^ Core Center for Molecular Morphology, Section for Stereology and Microscopy, Department of Clinical Medicine, Aarhus University and Department of Pathology, Aarhus University Hospital, Denmark, ^2^Department of Biomedicine, Aarhus University, and ^3^Centre for Stochastic Geometry and Advanced Bioimaging, Aarhus University, Wilhelm Meyers Allé 3, Building 1233/1234, 8000 Aarhus C, Denmark.

^4^Human Genetics, Faculty of Medicine, University of Augsburg, Stenglinstrasse 2, 86156 Augsburg, Germany

^5^Present address: Institute of Pathology, Hannover Medical School, Carl-Neuberg-Str. 1, 30625 Hannover, Germany.

^6^Present address: The Hormel Institute, University of Minnesota, Austin, Minnesota

^*^Correspondence to:

Email: [bsander@csgb.dk](mailto:bsander@csgb.dk)

Running title: Structural characterization of the PAR complex

**Table S1.** List of primer sequences used in this study.

| **No.** | **Primer name** | **Sequences (5’ 🡪 3’)** | **Purpose** |
| --- | --- | --- | --- |
| 1 | PAR6for1 | TAGAATTCATGGCCCGGCCGCAGAGGACTCCG | Amplification of full-length, human *PAR6* from cDNA |
| 2 | PAR6rev1 | GAAGTCGACTCAGAGGCTGAAGCCACTACCATCTCC |  |
| 3 | PAR6for4III | TTAGTCGACATGGCCCGGCCGCAGAGG | Subcloning of human *PAR6* into donor vector pIDC |
| 4 | PAR6rev4 | GCTCTAGATCAGAGGCTGAAGCCACTACCATCTC |  |
| 5 | PAR6for3II | AATCCATGGATGGCCCGGCCGCAGAGG | Subcloning of human *PAR6* into donor vector pIDS |
| 6 | PAR6rev3II | GGATTGCTAGCTCAGAGGCTGAAGCCACTACC |  |
| 7 | PKCfor1 | TAGAATTCATGCCGACCCAGAGGGACAGCAGC | Amplification of full-length *aPKC* from cDNA |
| 8 | PKCrev1 | GCCGTCGACTCAGACACATTCTTCTGCAGACATC |  |
| 9 | PKCfor2III | TTAGTCGACATGCCGACCCAGAGGGAC | Subcloning of human *aPKC* into donor vector |
| 10 | PKCrev2II | GCTCTAGATCAGACACATTCTTCTGCAGAC |  |
| 11 | HAtag_F | GATCCATGTACCCCTACGACGTGCCCGACTACGCCG | Replacement of 3×FLAG with HA tag of pPKC-8 |
| 12 | HAtag_R | CTAGCGGCGTAGTCGGGCACGTCGTAGGGGTACATG |  |
| 13 | PAR3for1 | TAAGTCGACATGAAAGTGACCGTGTGCTTCGGACGG | Amplification of full-length human *PAR3* from cDNA |
| 14 | PAR3rev1 | AGCAAGCTTTCAGGAATAGAAGGGCCTCCCTTTCTCAG |  |
| 15 | D70K_F | GACATTCTTTGTGATGTAGCAAAGGATAAAGACAGACTGGTAGCA | Introduction of D70K mutation in PAR3 |
| 16 | D70K_R | TGCTACCAGTCTGTCTTTATCCTTTGCTACATCACAAAGAATGTC |  |
| 17 | V13D_F | GACCCGGGTGGACGTGCCGTGCG | Introduction of V13D mutation in PAR3 |
| 18 | V13D_R | CGCACGGCACGTCCACCCGGGTC |  |

**Table S2.** List of plasmids constructed in this study.

| **Construct** | **Tag** | **Mutation** | | **Restriction enzyme site** | **Vector** | **Primer** |
| --- | --- | --- | --- | --- | --- | --- |
| **<*human PAR6A*> GenBank accession code: NP_001032358.1** | | | | | | |
| pPAR6 | none |  | | EcoRI-SalI | pUC57 | PAR6for1-PAR6rev1 |
| pPR6-6 | none |  | | SalI-XbaI | pIDC | PAR6for4III-PAR6rev4 |
|  | | | | | | |
| **<*human aPKC*> GenBank accession code: NP_002731.4** | | | | | | |
| pPKC | none | |  | EcoRI-SalI | pUC57 | PKCfor1-PKCrev1 |
| pPKC-5 | none | |  | SalI-XbaI | pIDC | PKCfor2III-PKCrev2II |
| pPKC-8 | 3×FLAG | |  | EcoRI-SalI | pGS-BacA-21122 | PKCfor1-PKCrev1 |
| pPKC-11 | none | | K283R | SalI-XbaI | pIDC | K283R_F-K283R_R |
| pPKC-12 | HA | |  | EcoRI-SalI | pGS-BacA-21122 | HAtag_F- HAtag_R |
|  | | | | | | |
| **<*human PAR3*>** | | | | | | |
| pPAR3 | none | |  | SalI-HindIII | pUC57 | PAR3for1-PAR3rev1 |
| pPR3-12 | 3×FLAG | |  | NheI-SphI | pGS-BacA-21122 |  |
| pPR3-15 | 3×FLAG | | V13D  /D70K | NheI-SphI | pGS-BacA-21122 | V13D_F-V13D_R/  D70K_F-D70K_R |

**Table S3.** EM datasets used for structural characterization of the PAR complex.

| **Sample** | **Mean defocus**  **(**$\boldsymbol{\pm}$ **s.d.) [Å]** | **No. of selected particles** | **Mean particles per class** |
| --- | --- | --- | --- |
| aPKC—PAR6 | 11556$\pm$5775 | 5954 | 23.8 |
| PAR3 _V13D,D70K_ | 18372$\pm$4027 | 12075 | 30.0 |


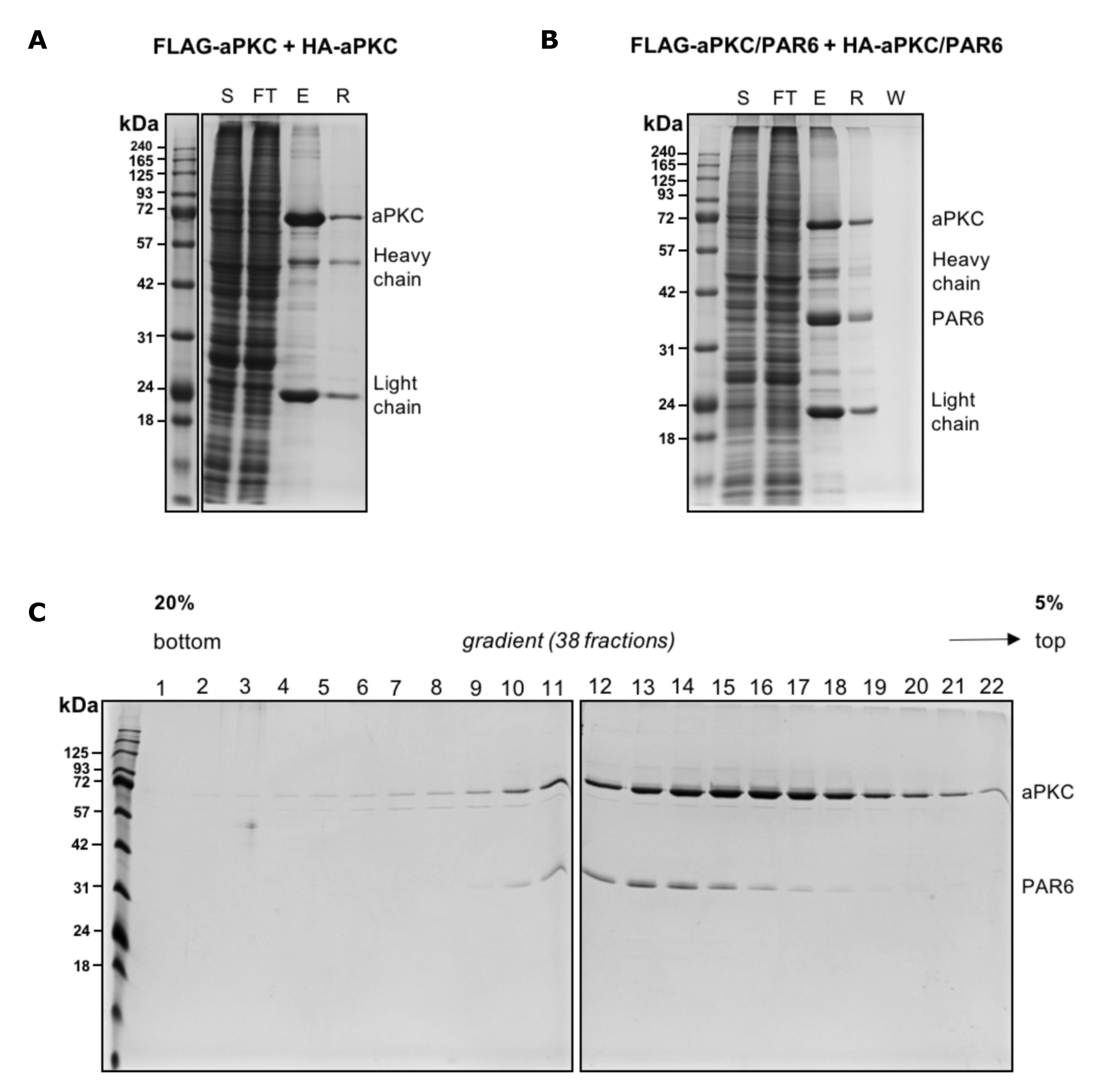


**Figure S1. Characterization of the aPKC/PAR6 complex. (A,B)** Either HA-aPKC and 3×FLAG-aPKC alone (A) or HA-aPKC and 3×FLAG-aPKC in combination with PAR6 (B) were co-expressed, pulled-down using anti-FLAG affinity purification and visualized by SDS-PAGE and Coomassie staining. Proteins were eluted by the SDS loading dye combined with heat denaturation. Proteins are indicated to the right. S, FT, W, E and R correspond to supernatant, flow through, last wash, eluate and the remaining FLAG resin denatured by SDS loading dye and heat treatment. **(C)** SDS-PAGE results of the aPKC/PAR6 complex eluted by 3×FLAG peptide and loaded on a 5-20% glycerol gradient are shown. Protein samples were collected in a total of 38 fractions. Fractions (1-22) are shown in the SDS-PAGE. Proteins and FLAG heavy chains and light chains are indicated to the right.
